# Using QI Methodology to Improve a Program’s QI Curriculum: An Educational Improvement Project

**DOI:** 10.1097/pq9.0000000000000598

**Published:** 2022-09-08

**Authors:** Courtney M. Port, Kathleen M. Donnelly

**Affiliations:** From the Department of Pediatrics, Inova Children’s Hospital, Falls Church, Va.

## Abstract

Quality improvement (QI) as a method of obtaining meaningful change is increasingly valued. A few comprehensive, longitudinal curricula demonstrate efficacy, patient impact, and behavior change over time. This educational improvement study aimed to create a curriculum that increased resident proficiency in practicing QI principles, score on the QI Knowledge Application Tool-Revised, and QI projects completing at least 2 plan-do-study-act (PDSA) cycles in 5 years.

**Methods:**

We utilized The Model for Improvement and sequential PDSA cycles, testing curricular components for improvement. Measures were analyzed annually (2014−2020). The curriculum includes modules and didactic workshops for foundational knowledge, rapid personal improvement projects for putting knowledge into practice, and experiential learning through developing and leading QI projects.

**Results:**

Graduating residents reporting proficiency in practicing QI principles increased from 4 (44%) to 11 (100%). The average QI Knowledge Application Tool-Revised score increased from 50% to 94% (95% CI, 37–51). Resident QI projects completing at least 2 PDSA cycles increased from 30% (n = 3) to 100% (n = 4), *P* = 0.0005, while projects achieving improvement increased from 40% (n = 4) to 100% (n = 3), *P* = 0.002. Patients were also positively impacted, with 63% (n = 3) of clinical QI projects that measured patient-centered outcomes achieving improvement and 69% (n = 11) of clinical QI projects improving clinical processes.

**Conclusions:**

This study developed a curriculum that successfully prepares residents to practice QI principles and lead multidisciplinary QI projects while demonstrating patient impact and behavior change. It offers an example of curriculum development and evaluation aided by QI science.

## INTRODUCTION

Quality improvement (QI) science is increasingly valued as a method for obtaining meaningful change. From the oath to “do no harm” to increasing requirements for QI skills and reliance on quality indicators for reimbursement, improving health care quality is a focus for practitioners, educators, and administrators. Yet, resident physician QI education reviews have shown variability in educational components, modalities, and duration.^1,2^ The 2016 Clinical Learning Environment Review (CLER) National Report^3^ found that despite significant progress, much work remains, especially when it comes to meaningful engagement. Most clinical learning environments had few residents with working knowledge of QI. In describing resident participation in QI projects, only a limited number of programs had residents “designing, managing, or analyzing the results of the activity.” Rather, implementing a hospital-wide initiative such as hand hygiene was common. The experiential learning necessary for professional development is lost when trainee involvement is only peripheral in QI work.^4^

Evaluation of educational efforts is also frequently discussed as a significant QI educators’ challenge.^1,2,5,6^ Previous reviews showed that most studies were before–after designs,^2,7^ and a few programs measured clinical impact.^1,5^ Additionally, in a 2021 realist synthesis, only 11% (n = 18) of the postgraduate medical education QI curricula were in pediatrics, and only 0.9% (n = 2) of the studies were designed as QI reports.^8^ In the most recent systematic review, authors found that although the number of published studies increased over time, the number reporting clinically relevant outcomes decreased despite an increase in studies requiring QI project completion.^7^ Although most curricula contain an experiential component,^4^ many programs continue to wrestle with simultaneously optimizing learning and project outcomes within complex health care settings.^4^

QI is a core competency.^9,10^ Education for physicians must be effective, emphasizing progressive learning goals over the length of residency and not just a few isolated learning experiences. Although the literature supporting QI education is growing, there are few comprehensive and longitudinal curricula demonstrating efficacy, patient impact, and future behaviors.^1,2,5,7^ Thus, curricula that achieve progressive learning goals may be more valuable to programs striving to improve QI education than descriptions of any particular curricular element.

This educational improvement study aimed to demonstrate a comprehensive, longitudinal curriculum that increased the percentage of residents reporting proficiency in practicing QI principles, scoring 90% or greater on the QI Knowledge Application Tool-Revised (QIKAT-R),^11^ and completing QI projects with at least 2 plan-do-study-act (PDSA) cycles to 90% within 5 years.

## METHODS

### Context

This single-center study occurred in a 226-bed children’s hospital within an independent academic medical center. The institution’s pediatric residency trains 13 categorical residents per year. Before this curriculum’s implementation, residents received no formalized QI training. Trainees were directed to identify an area for improvement within their work environment and work toward improvement as time allowed with no required level of completion. No faculty had been formally trained in QI at this curriculum’s initiation. The residency program currently includes 22 core faculty and 1.65 full-time-equivalent positions for education and scholarly activity, with 0.2 full-time-equivalent specifically dedicated to quality and safety education.

### Needs Assessment

The residency program did not have a QI curriculum at this study’s initiation. The authors completed a needs assessment, utilizing QI and patient safety (PS) content from the CLER Pathways to Excellence,^12^ American Board of Pediatrics general pediatrics content outline^13^ and Maintenance of Certification (MOC) part IV specifications,^14^ and Accreditation Council for Graduate Medical Education Common Program Requirements^15^ to generate themes. The authors then listed possible education modalities and created corresponding measures for each item to track compliance and determine the curriculum’s success (**Table 1, Supplemental Digital Content,** http://links.lww.com/PQ9/A407).

### Rationale

Improvement science offers several benefits to the evaluation and improvement of medical education, including focusing on the local educational setting, integrating a deep understanding of the local context, and testing multiple hypotheses and interventions over time.^16^ The Standards for Quality Improvement Reporting Excellence in Education standardized this approach in 2019.^16^

### Interventions

The curriculum was first implemented during the 2014−2015 academic year and progressed through several iterations in response to feedback from trainees and process improvement efforts to achieve stated goals. The current curriculum is a 3-year, longitudinal progressive education (Table 1). Online QI modules through the Institute for Healthcare Improvement (IHI)^17^ and seven-to-ten 50-minute didactic workshops create foundational knowledge, followed by rapid personal improvement projects to put knowledge into practice during the intern year. The curriculum culminates in developing, leading, and completing multidisciplinary group QI projects during the second and third postgraduate years. In addition, each resident performs a root cause analysis (RCA) on an actual PS event involving a patient cared for by residents and presents their findings at a quarterly PS conference.

**Table 1.** Curricular Content Included in the 3-Year Program

Didactic Content		Experiential Content
Quality Improvement	Patient Safety	QI and PS
Intern Year		
IHI QI modules	Institutional priorities	Develop and complete a personal improvement project
QI definitions	PS definitions	
QI versus traditional research^*^	Human factor design	Identify a quality gap
QI methodologies: lean, six sigma, The Model for Improvement	Case-based workshop^*^	Create a driver diagram
	Define errors	Create a SMART aim
	Identify cognitive biases	
The Model for Improvement^*^		
Build a SMART aim	Identify system failure modes	Develop measures
Develop measures		
Measurement journey	Propose system-based solutions	Develop change ideas
Operational definitions		
	Epidemiology of PS	
Performing PDSA cycles	Prevalence of errors	Test changes in PDSA cycles
Analyzing a run chart	Types of PS errors	
	Use of trigger tools	
	Error prevention	
	Real time: psychological safety	
	Future patients: PS reporting	
Understanding variation		
Creating QI tools^*^		
Process map, Fishbone and driver diagrams, and Pareto chart		
Change concepts		
Statistical process control charts		
		Track measures on run chart
		Analyze result of run chart
		Present your QI project
Second and Third Year Combined		
Presenting group QI project updates (2 sessions/y)	Failure effects mode analysis^*^	Develop and complete a group QI project (≥ 2 PDSA cycles and Act as QI lead >3–4 blocks)
		Perform a RCA for the Quarterly Patient Safety Review
All 3 Years Combined		
2	Annual RCA Workshop^*^	Attend hospital or department RCAs
	Diagnostic errors, overutilization, and overdiagnosis	
		Attend system, hospital, and/or department quality and safety committee meetings
	Quarterly Patient Safety Review^*^	
	Resident run RCA	

There are seven to ten 50-minute sessions for interns and three 50-minute sessions for seniors during academic half-day didactics. There is no dedicated nor protected time for the experiential content. IHI QI modules are completed on the resident’s own time and due before each academic half-day QI session.

^*^Workshops.

#### Study of the Interventions

Scheduled resident feedback sessions provided detailed reflections as PDSA cycle-level data for each test of change. Curricular components were modified based on this feedback and progress on study measures.

#### Evolution of Interventions over Time

Several curricular components evolved over time (Table 2). Initially, the curriculum director taught core concepts in lecture format. However, residents reported difficulty keeping up at subsequent lectures due to inconsistent attendance. The following year, we required completion of online IHI QI modules and created supplemental workshops to review and practice implementing QI tools. Workshops included sessions on error classification and system-based solutions for PS events; creating a detailed process map crossing several disciplines and containing multiple levels of decision branching; creating key driver and fishbone diagrams; and developing and interpreting run charts using gamification, which is the process of adding games or game-like elements to a task. Attendance at QI sessions improved when didactics changed to a single academic half-day schedule.

**Table 2.** Iterative Changes to a Quality Improvement Curriculum, 2014–2020

Year	Changes	Outcomes	Lessons Learned
2014	Curriculum initiated with core lectures for foundational knowledge	Poor attendance	Ensure all residents receive content for foundational knowledge
		Difficulty understanding content of subsequent lectures by those who were absent	
2015	Foundational knowledge from core lectures replaced with online IHI QI modules	Residents felt the IHI QI modules were beneficial	Assign IHI QI modules over shorter time frame
		Timing of completion of individual modules varied and was too spread out	Supplement modules with workshops
Late 2015	IHI QI Modules supplemented with workshops to practice using tools and applying concepts	Workshops provided insight into local systems and interdisciplinary roles	Continue workshops
		2016 QIKAT-R scores increased from PGY1 to PGY2, but similar gains were not obtained in PGY3	Shift focus to improve experiential learning curricular components during late PGY1 and into PGY2
2016	PGY1 rapid personal improvement projects were changed from group to individual projects to ensure all residents have a basic understanding of the components of a QI project	Residents found the personal improvement project a good learning experience, but too time-consuming	Provide more guidance on achievable aims to avoid burdensome data collection
		Residents reported difficulty choosing measures and results display	Schedule sessions for feedback on project proposals before baseline data collection
		2017 QIKAT-R scores improved to goal for PGY3s	Shift focus to success of PGY2-3 group QI projects
2017	The program requirement was changed such that each resident must participate in 1 of 3–4 group projects in their PGY2–3 year to improve project rigor	Residents expressed difficulty with team functioning while on different rotations	Ensure group projects are progressing each block
		2017 group QI projects were still not completing minimum of 2 PDSA cycles and only 50% are achieving success	
2018	Instituted regularly scheduled check-ins with each QI team to monitor progress over time	Residents reported unfair distribution of work on some projects	Ensure each resident takes the project lead role during 2–3 block rotations per year
		2018: 100% of group QI projects completed 2 PDSA cycles and 100% of PGY3s reported proficiency in practicing QI principles	
2019	Added a resident QI project lead (self-selected) to each block rotation with clear expectations and responsibilities, including meeting with QI director for coaching and accountability	Some residents reported improved distribution of work and experiential learning on group projects	Provide consistent meetings to increase accountability of all resident QI project leads
		Project abstract accepted at a national conference and later for publication in peer-reviewed journal	Increase flexibility of meetings
			Start group projects sooner to ensure postintervention data available for conferences
		Heavy burden placed on QI director led to inconsistent meetings	
			Improve faculty QI knowledge
2020	Tool developed to track meetings. Virtual option added. IHI modules completed during intern orientation, allowing earlier initiation of projects. Six faculty development QI sessions for PHM	Most residents reported improved distribution of work and experiential learning on group projects	Increase interdisciplinary involvement in group projects
		Increase in national conference presentations	Improve balance between institutional goals and resident interest for project topics
		Improved consistency of meetings with QI director	Focus on QI topics with poor self-reported performance (such as FMEA)

FMEA, failure mode and effects analysis; PGY, postgraduate year; PHM, Pediatric Hospital Medicine.

Initially, residents completed personal improvement projects in groups with a shared aim. This approach led to disparate knowledge and disjointed projects that did not achieve their aims. By the curriculum’s third year, residents completed personal improvement projects individually to consistently gain the necessary foundational knowledge.

We then turned our focus to resident QI projects. Unfortunately, the overall quality and success of projects were low. Residents were passionate about their project of choice but did not have the time to complete them independently. In addition, project faculty mentors lacked QI education and experience. To provide more mentoring and coaching by the quality curriculum director, rather than completing independent projects, each resident was required to participate in 1 of 3–4 group projects per class, reducing the total number of QI projects per year.

Subsequently, some residents expressed frustration from poor team dynamics and unfair distribution of tasks. In response, team members were asked to assign rotating resident QI project leads for each academic block. Responsibilities included meeting with the curriculum director for coaching and accountability, completing project tasks such as data collection and analysis, and communicating project updates to other project team members. This increased transparency such that less engaged residents may improve their participation. Open to all project team members, these meetings were frequently one-on-one due to busy resident schedules. The agenda consisted of project status within its timeline, results, barriers to success, and concrete “to do” tasks for that academic block. The curriculum director and resident QI lead discussed team dynamics and conflict resolution strategies when necessary. Informal handoff occurred between QI lead residents by email each block.

### Measures

We borrowed the primary outcome measure from the Association of American Medical College’s aim in Teaching for Quality,^9^ the percentage of graduating residents self-reporting “proficiency in practicing QI principles in their everyday work.” Data were collected annually via a local survey in which positive answers included agree and strongly agree. We chose this as the primary outcome measure because one’s level of proficiency encompasses more than any single objective assessment, and the perception of one’s proficiency is an important driver of future QI endeavors. We balanced this self-reported proficiency with other objective measures of skill, including the percentage of graduating residents scoring >90% (24 out of 27 points) on the QIKAT-R, which tests residents’ ability to develop an aim statement, define measures, and propose system-level changes for 3 cases. The QIKAT-R has been validated in previous studies.^11^ The curriculum director administered and scored the QIKAT-R at the end of each academic year. A supplementary outcome measure was the percentage of resident QI projects that achieved improvement.

Process measures included attendance, participation in curricular components, and the percentage of resident QI projects completing at least 2 PDSA cycles. Since the educational activities of this curriculum occupied finite didactic time in the academic schedule, we tracked the American Board of Pediatrics board passage rate as a balancing measure. The 3-year pass rate was chosen to counter expected year-to-year variability. In addition, we emailed an alumni survey in 2019 to 49 graduates who had completed the curriculum since 2014 and for whom we had current email addresses to ascertain sustainability of improvement and to understand the application of QI skills after graduation.

The authors evaluated several measures over time to assess resident behavior changes, including self-reporting the number of PS events reported and medical errors disclosed. Moreover, during the RCA investigation, the curricular director evaluated each resident’s ability to elicit system-based causes and solutions. Additional measures extending beyond residents were impacts on faculty (number participating in resident QI initiatives and obtaining MOC part IV credit), the greater academic community (the number of conference presentations and publications), and patients (percentage of clinical projects achieving improvement in health care processes or patient-centered outcomes).

### Analysis

We collected annual prospective resident survey data, including the QIKAT-R, self-reported proficiency in practicing QI principles, the number of PS events reported and medical errors disclosed, beliefs regarding QI work, and satisfaction with the curriculum. Resident QI scholarly activity was tracked for quality and quantity. The Model for Improvement and sequential PDSA cycles were utilized, testing curricular changes for meaningful improvement in desired outcomes. Measures were plotted graphically by year. Due to the small number of data points, statistical process control charts were not used. Two-tailed Fisher exact tests and Mann-Whitney U tests determined whether significant differences were present between groups.

### Ethical Considerations

This study was a QI activity and not human subject research. Therefore, review and approval by the local institutional review board were not required.

## RESULTS

**Fig. 1.**
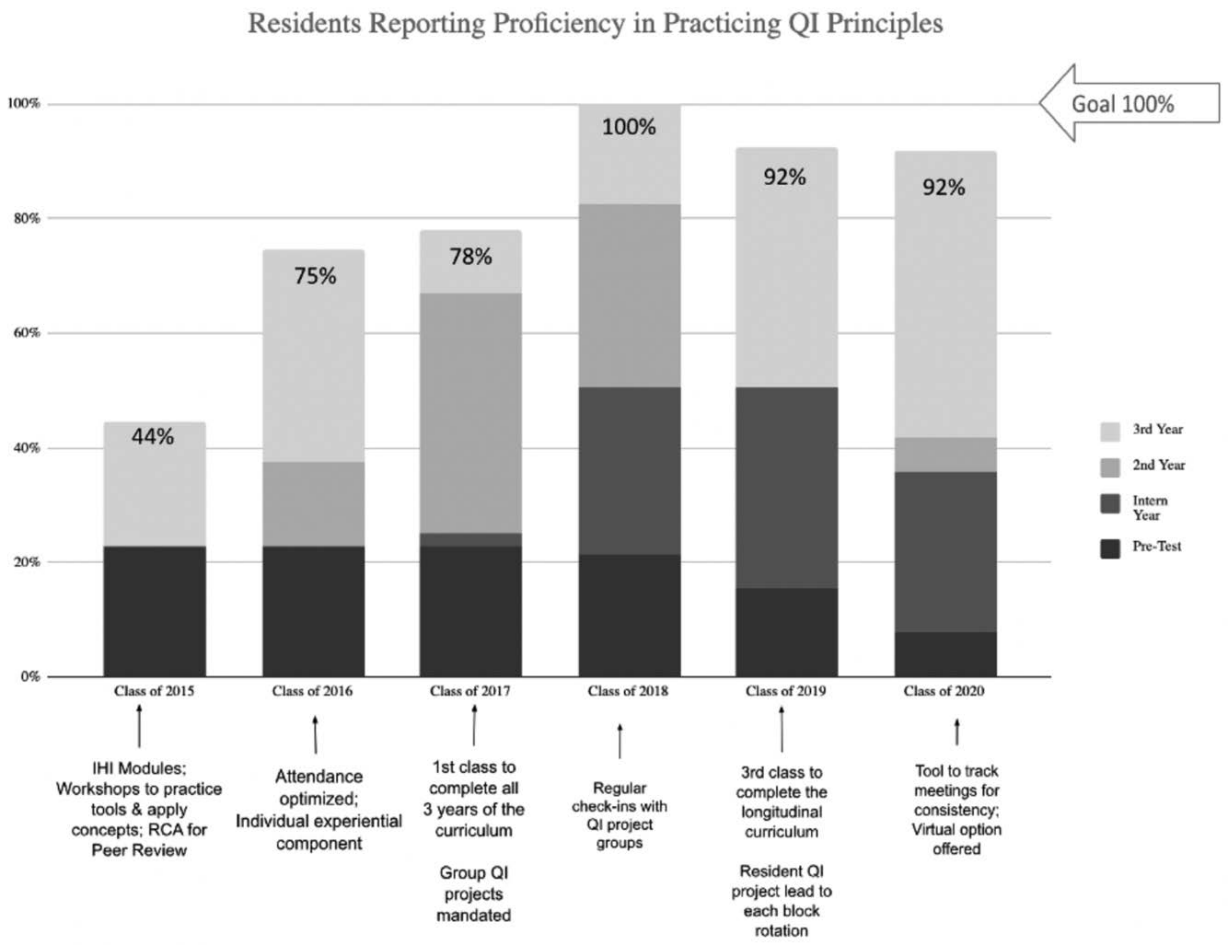
Percentage of residents reporting proficiency in practicing QI principles in the context of their everyday work. The proportion of residents in the class of 2015 compared with the class of 2020 who reported proficiency was significant, *P* = 0.046, by Fisher exact testing. Note: posttests were administered at the end of each academic year.

**Fig. 2.**
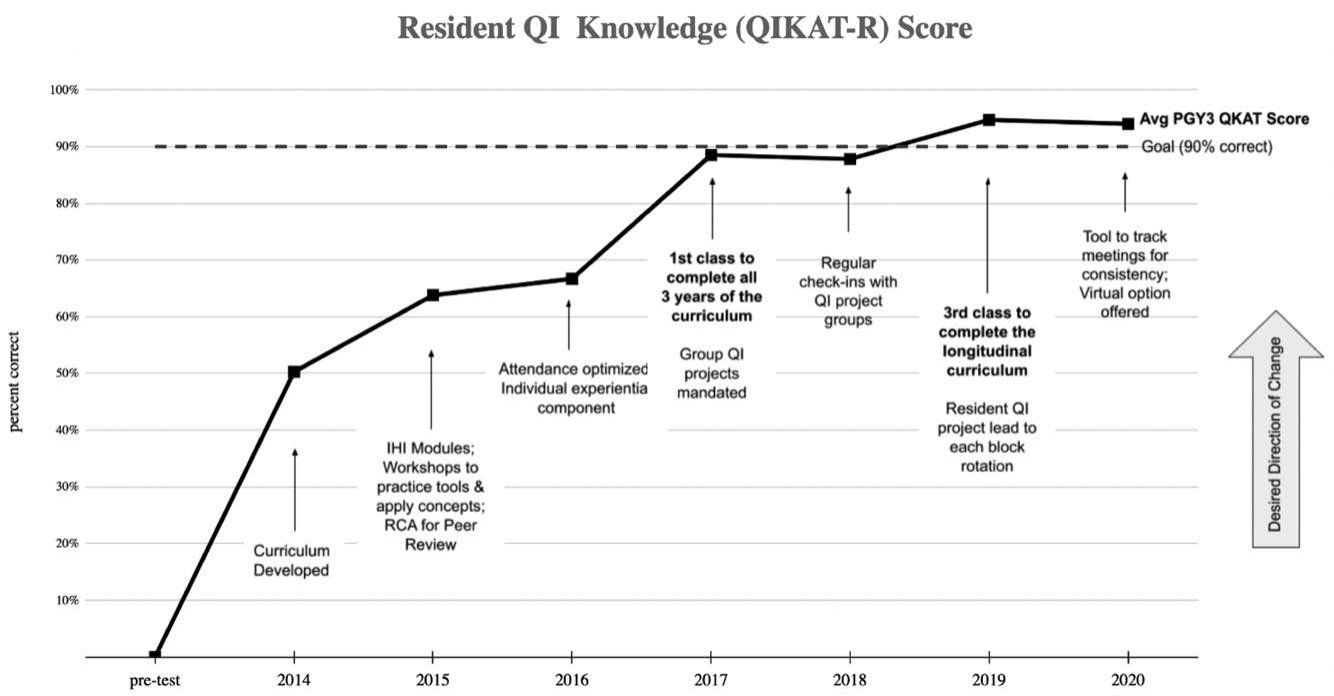
Average QI knowledge application tool-revised score for graduating residents by year. Run chart with y-axis demonstrating the average percent correct on the QIKAT-R over time. Scores are represented as percentages correct out of a possible 27 points. PGY indicates postgraduate year.

The study aimed to increase the percentage of residents reporting proficiency in practicing QI principles, scoring *>*90% on the QIKAT-R, and completing QI projects with at least 2 PDSA cycles to 90% within 5 years. An average of 90% of residents completed the annual QIKAT-R and survey, ranging from 69% to 95% and remaining at >90% for the last 3 years. Within 4 years, and by the second graduating class receiving the entire longitudinal curriculum, residents reporting proficiency in practicing QI principles increased from 4 (44%) to 11 (100%) per year (Fig. 1). Those reporting proficiency before the curriculum implementation, 23% (n = 8), compared with those graduating in 2020, 92% (n = 12), was significant (*P* = 0.0001). Average graduating resident QIKAT-R scores increased from 0% before curriculum implementation to exceeding the goal of an average of 90% correct (*P* < 0.0001) (Fig. 2). Table 3 summarizes the results by Kirkpatrick model^18^ category, a widely accepted framework for evaluating educational interventions: (1) participation and reactions, (2) knowledge, skills, and attitudes, (3) behavioral change, and (4) change in organizational practice and demonstration of benefit to patients.

**Table 3.** Kirkpatrick Model of Evaluation Levels with Associated Specific Metrics and Results for Quality Improvement Curriculum Evaluation

Kirkpatrick Model of Evaluation		QI Curriculum Evaluation Metrics	Pre/Early Curriculum	Post curriculum		*P*
1	Participation	% Attendance/participation in curricular components^*^	n/a	100% (n = 13)		—
	Satisfaction	% Satisfied with curriculum	n/a	96% (n = 50)^†^		—
2	Attitudes	% Reporting proficiency in QI skills	23% (n = 8)	92% (n = 12)		0.0001
		% Perceiving QI as essential to providing good patient care^‡^	80% (n = 28)	100% (n = 13)		0.17
	Knowledge	% Scoring ≥90% on QIKAT-R	0% (n = 0)	85% (n = 11)		<0.00001
	Skills	% QI projects completing ≥2 PDSA cycles	30% (n = 3)^§^	100% (n = 3)		0.0005
		% Residents completing RCAs on PS events	0% (n = 0)	100% (n = 13)		<0.00001
3	Behavioral change	Average no. PS reports	2–5 per resident^¶^	2–5 per resident		—
		Average no. medical errors disclosed to patients	1 per resident^¶^	2–4 per resident		—
		% Residents utilizing system-based solutions during RCAs	0% (n = 0)^¶^	100% (n = 13)		0.001
		No future QI projects by residents after graduation	unknown	14^†^		—
4	Change in organizational practice	% QI projects achieving success	40% (n = 4)^§^	100% (n = 3)		0.002
		% Clinical QI projects achieving improvement in health care processes	10% (n = 1)^§^	69% (n = 11)^†^		0.001
		No. QI projects completed in the local healthcare system	10^§^	31^†^		—
		No. faculty participating in resident QI initiatives and obtaining MOC part IV credit	0^§^	32^†^		—
		No. conference presentations at medical conferences	10^§^	33^†^		—
		Demonstration of benefit to patients	% of clinical QI projects achieving improvement in patient-centered clinical outcomes	10% (n = 1)	60% (n = 3)^c^	0.036
	Balancing measure	3-y average general pediatrics certifying exam passage rate for first-time test takers	82% (n = 29)	97% (n = 38)		0.11

Where possible, metrics compare precurriculum data with the most recent postcurriculum (2020) data. However, certain data were not collected before curriculum implementation. In these instances, we present the earliest data collected.

^*^IHI modules; workshops on process maps, driver and fishbone diagrams, medical error classification and system-based solutions, run chart and statistical process control chart; personal improvement projects; RCA performance; group QI project leadership role.

^†^Total data available since initiation of the curriculum. Two-tailed Fisher exact tests determined if significant differences were present between groups.

^‡^Essential to: patient outcomes; personal performance; safe, effective, efficient, equitable, time-sensitive, and patient-centered care.

^§^Data first collected at the end of the 2015 academic year.

^¶^Data were first collected at the end of the 2017 academic year. All results are from the graduating class unless otherwise specified.

Although resident projects decreased from 10 in 2015 to 3–4 per year, project rigor and success improved. The proportion of resident QI projects completing at least 2 PDSA cycles increased from 30% (n = 3) in 2015 to 100% (n = 4) by 2018, *P* = 0.0005. In addition, the percentage of projects achieving improvement was 40% (n = 4) in 2015 and reached 100% (n = 3) by 2019, *P* = 0.002. Table 4 demonstrates curricular impact over time.

**Table 4.** Table of Curriculum Outcomes over Time

Measure	2014 Pretest	2015	2016	2017	2018	2019	2020	*P*
Graduating residents reporting proficiency in practicing QI principles	23% (n = 35)	44% (n = 9)	75% (n = 5)	78% (n = 9)	100% (n = 12)	92% (n = 13)	92% (n = 13)	0.0001^*^
Graduating residents achieving 90% correct (score of 24 and higher) on the QIKAT-R	0% (n = 35)	0% (n = 9)	0% (n = 5)	56% (n = 9)	67% (n = 12)	100% (n = 13)	85% (n = 13)	<0.0001^*^
Average graduating resident class QIKAT-R % correct score	50%(n = 35)	64% (n = 9)	67% (n = 5)	89% (n = 9)	88% (n = 12)	95% (n = 13)	94% (n = 13)	<0.0001^†^
Percentage of graduating resident QI projects completing at least 2 PDSA cycles	—	30% (n = 10)	57% (n = 7)	75% (n = 4)	100% (n = 3)	100% (n = 3)	100% (n = 3)	0.0005^*^
Percentage of graduating resident QI projects achieving improvement	—	40% (n = 10)	43% (n = 7)	50% (n = 4)	67% (n = 3)	100% (n = 3)	100% (n = 3)	0.002^*^

Parentheses note total number (denominator).

^*^P value determined using 2-tailed Fisher exact testing.

^†^*P* value determined using the Mann-Whitney U test.

Notably, patients were also positively impacted. Sixty percent (n = 3) of clinical QI projects that measured patient-centered outcomes achieved improvement, and 69% (n = 11) of clinical QI projects improved clinical healthcare processes (**Table 2**, **Supplemental Digital Content,** http://links.lww.com/PQ9/A407). The success of resident education in QI science was not at the expense of other core didactics, as represented by our balancing measure, the 3-year average general pediatrics certifying exam passage rate for first-time test takers: 82% (n = 29) for 2012−2014 and 97% (n = 38) for 2018−2020. Additionally, from 2015 to 2020, this curriculum added 31 QI projects to our local healthcare system, 1 journal publication, 33 presentations at medical conferences, and MOC part IV credit for 19 faculty and 40 residents. In addition, this curriculum provided 22 faculty with experiences as QI team members, learning the application of methodology and tools alongside residents.

PS reporting of medical errors by graduating residents remained the same atan average of 2–5 reports per resident in 2017 when these data were first collected and again in 2020. However, residents disclosing medical errors to patients increased with an average of 1 disclosure per graduating resident in 2017 compared with 2–4 disclosures in 2020. Within the last graduating class, all residents submitted multiple PS reports and disclosed an error at least once, with 92% (n = 12) disclosing errors more than once. Residents also increasingly identified system-based solutions during RCA investigations [0% in 2017; 100% (n = 13) in 2020]. Ninety-four percent (n = 32) of residents receiving the full 3-year curriculum were satisfied with the QI training they received during residency, and none were dissatisfied. Twelve alumni reported completing 14 additional QI projects, and 1 graduate commented that the curriculum “sparked an interest, and I have since completed a fellowship in QI.” Another expressed having “much more knowledge of QI compared with my cofellows.”

## DISCUSSION

This study developed a comprehensive, longitudinal QI curriculum that effectively established resident knowledge, comfort, and QI skills through careful measurement and iterative change, but importantly also benefited patients and impacted resident behaviors. The specific aims were met, resulting in a significant increase in the percentage of residents reporting proficiency in practicing QI principles, scoring >90% on the QIKAT-R, and completing QI projects with >2 PDSA cycles to 90%.

The percentage of graduating residents reporting proficiency in practicing QI principles was not sustained at 100%, dropping to 92% the following 2 years. This decrease was possibly due to common cause variation. However, we theorized that poor distribution of participation within the group QI projects contributed. To address this, we adjusted the workflow among group QI project members, designating each resident as project lead for at least 3 educational blocks per year. We have not yet fully evaluated this adjustment.

The change in the number of resident QI projects from 10 to 3–4 per year may have had the greatest impact on the rigor and success of projects—fewer projects allowed for more frequent coaching from the curriculum director. In addition, the group rather than individual projects allowed for shared responsibilities during busy clinical rotations. Therefore, we recommend reducing the number of QI projects per year for programs with limited QI-trained faculty. This recommendation is consistent with findings by Brown et al^8^ that team-based, heavily mentored projects with frequent meetings are associated with successful curricula. While group projects have the potential for workload maldistribution and making individual resident assessment difficult, scheduled meetings with assigned resident leads allow for individual knowledge and participation evaluation.

Our QI curriculum shares elements associated with successful curricula, including longitudinal curricula with experiential, project-based learning with topics screened to ensure appropriate scope and alignment with health system priorities; frequent meetings to maintain momentum; clear expectations and deliverables; and academic incentives for faculty.^8^ Also, our study demonstrates a successful curriculum despite lacking protected time for projects and easily accessible data.

This educational improvement study also offers a method by which curriculum development can be aided by QI science, creating successful content and measuring and achieving specific outcomes over time. The next steps in this study will involve targeting QI skills with poor performance and increasing faculty development in QI science.

### Strengths

Strengths of the curriculum include the continuous analysis and modification of the content, utilization of free educational resources, and robust experiential learning opportunities. In addition, there were many facilitators to our success. Most importantly, there has been strong programmatic support, dedicating time within the didactic schedule, and funding for the director role. This role provided time to oversee assignments, facilitate workshops, mentor and coach projects, and evaluate the curriculum. Results may be generalizable to similarly sized programs with leadership buy-in and funding for the director position. Training for the curriculum director role was self-directed and did not require additional graduate degrees.

### Limitations

There were several limitations to this study. First, the small number of participants in this single-center study will limit the generalizability of the results. Second, some data were not collected before the initial implementation of the curriculum, limiting the comparisons without a true baseline. Third, data for many measures, including the primary outcome measure, were self-reported, which may be exaggerated due to social desirability bias. Future research should focus on effective workplace assessment of proficiency in practicing QI principles in everyday work. Finally, the longitudinal nature of the curriculum limited the ability to adjust interventions rapidly.

## CONCLUSIONS

This study successfully developed a comprehensive and longitudinal QI curriculum that demonstrates efficacy and positive impact on patient and resident behaviors while preparing residents to practice QI principles and lead multidisciplinary QI projects. It also demonstrates how QI science aids curriculum development and evaluation.

Presented at the American Academy of Pediatrics National Conference, October 2019, New Orleans, La. and the Pediatric Hospital Medicine Annual Conference, July, 2017, Nashville, Tenn.

## DISCLOSURE

The authors have no financial interest to declare in relation to the content of this article.

## Supplementary Material


