# Supplementary material for: Using QI Methodology to Improve a Program’s QI Curriculum: An Educational Improvement Project
Source: Pediatr Qual Saf. 2022 Sep 8;7(5):e598. doi: 10.1097/pq9.0000000000000598 (PMC9762927; doi:10.1097/pq9.0000000000000598)
Supplement: Supplementary file 1 [file pqs-7-e598-s001.pdf]

Supplemental Table 1: *Summary of Quality Improvement and Patient Safety Curriculum Requirements and Recommendations*

CLER Guidelines	Associated ABP Content Specifications, MOC Part IV Specifications, and/or RRC Guidelines	Educational Modality & Outcomes Measurement
PS Pathway 1: Reporting of adverse events, close calls (near misses) Reporting is an important mechanism to identify patient safety vulnerabilities. A robust reporting system is essential for the success of any patient safety program.<br><br>**Properties include:**<br>● Residents, fellows, faculty members, and other clinical staff members (nurses, pharmacists, etc.) know how to report patient safety events at the clinical site. *The focus will be on the proportion of individuals who know how to report.*<br>● Residents, fellows, faculty members, and other clinical staff members know their roles and responsibilities in reporting patient safety events at the clinical site. *The focus will be on the proportion of individuals who know their roles and responsibilities in reporting.*<br>● Faculty members report patient safety events via the clinical site's preferred system. *The focus will be on the proportion of faculty members who report safety events.*<br>● Residents/fellows report patient safety events via the clinical site's preferred system. *The focus will be on the proportion of residents/fellows who report safety events toward the goal of disseminating best practices and lessons learned across nearly all residency programs.*<br>● Patient safety events reported by faculty members and residents/fellows are aggregated into the clinical site's central repository for event reporting. *The focus will be on whether safety events, reported via any mechanism (e.g. online, telephone calls, reports to the department chain of command, morbidity and mortality reviews, claims committee) are captured in the site's central repository*.	ABP Content Specifications<br>**Definitions used in discussions of patient safety**<br>1. Understand and apply the definition of a medical error<br>2. Understand and apply the definition of a near-miss event<br>3. Understand and apply the definition of a sentinel event<br>4. Understand and apply the definition of a preventable adverse event<br>5. Understand and apply the definition of a non-preventable adverse event<br>**Detecting and reporting adverse events**<br>1. Understand the relationship between the detection of a medical error and the ability to discover and effect improvements<br>2. Identify barriers to reporting adverse events<br>3. Apply effective strategies to improve reporting of adverse events<br>4. Apply voluntary systems for reporting of adverse medical events<br>5. Recognize the use of National Patient Safety Goals to improve patient safety<br><br>**RRC PS/QI Guidelines**<br>VI.A.6. Residents and faculty members must demonstrate an understanding and acceptance of their personal role in the following:<br>● assurance of the safety and welfare of patients entrusted to their care	**Educational Modality**<br>1. QI Project Simulation Workshop: Increasing Resident Reporting of PS events (Identify barriers, Educate on Importance, Educate on what/how to report, Develop feedback mechanism to those reporting, Review support for those involved in PS events)<br>2. Resident Peer Review on Safety Events (each event is classified- ie preventable adverse event; abbreviated RCA is completed; recommendations for system-based solutions are made)<br>3. Didactic Lecture or IHI Module on PS Definitions<br><br>**Outcomes Measurement**<br>1. % Residents That Know How to Report PS Events<br>2. % Residents Who Know Their Roles & Responsibilities in Reporting PS Events<br>3. Average Number of Safety Events Reported by Residents<br>4. % PS Questions on In-service Exams Answered Correctly<br>5. % Residents Completing an Abbreviated RCA on a PS Event for Resident Peer Review
PS Pathway 2: Education on patient safety<br>Formal educational activities that create a shared mental model with regard to patient safety-related goals, tools, and techniques are necessary for health care professionals to consistently work in a well-coordinated manner to achieve patient safety goals.<br><br>**Properties include:**<br>● Residents/fellows receive patient safety education that includes	**ABP Content Specifications**<br>**Methods to reduce medical adverse events**<br>1. Recognize the relative role of systems and individuals in producing medical error and harm<br>2. Recognize what interventions can reduce error in situations (eg, stress, fatigue, distraction) at high risk for medical error<br>3. Understand the role of ancillary services such as the	**Educational Modality**<br>1. PS Didactics<br>2. Resident participation on hospital-wide interprofessional PS/QI committees with formal process for distribution of information to the residency program

information specific to the clinical site. *The focus will be on educational content directly related to the site's processes for ensuring the safety of its patient population, and on progress from basic training received early in the education process to basic training supplemented with periodic, interprofessional/team training educational experiences.*<br>● Faculty members are proficient in the application of principles and practices of patient safety. *The focus will be on the proportion of faculty members who report to be proficient in the application of principles and practices of patient safety at the clinical site.*<br>● Residents/fellows are engaged in patient safety educational activities where the clinical site's systems-based challenges are presented, and techniques for designing and implementing system changes are discussed. *The focus will be on the proportion of residents/fellows who are engaged in safety educational activities that include the above elements in their content—toward the ultimate goal of learning that is shared across programs.*<br>● Residents/fellows and faculty members receive education on the clinical site's proactive risk assessments (e.g., failure mode and effects analysis). *The focus will be on the proportion of individuals who receive education on this specific element.*<br>● The clinical site's patient safety education program is developed collaboratively by patient safety officers, residents/fellows, faculty members, nurses, and other staff members to reflect the clinical site's patient safety reporting processes, risk mitigation systems, experience, and goals. *The focus will be on the inclusion of GME leadership, residents/fellows, and faculty members in the process of developing the clinical site's patient safety education program and its dissemination throughout the organization, including to residents/fellows and faculty members.*	pharmacy in the prevention of medication errors<br>4. Understand the role of computerized order entry and dose-range checking in reducing medication errors<br>5. Understand the impact of product naming and packaging on medication safety<br>6. Understand the role of medical device design in prevention of medical error<br>7. Understand the contribution of patient factors to adverse events<br>8. Understand the role of patients and their families in reducing adverse events<br>**Key principles of patient safety**<br>1. Understand the importance of assessment and redesign of health-care processes before error occurs<br>2. Understand the importance of creating and maintaining a learning environment (eg, morning report, meetings with partners) in improving patient safety<br><br>**RRC PS/QI Guidelines**<br>**Systems-based Practice**<br>IV.A.5.f) Residents are expected to:<br>● advocate for quality patient care and optimal patient care systems;<br>● work in interprofessional teams to enhance patient safety and improve patient care quality;<br>● participate in identifying system errors and implementing potential systems solutions;<br>● advocate for the promotion of health and the prevention of disease and injury in populations<br>VI.A.3. The program director must ensure that residents are integrated and actively participate in interdisciplinary clinical quality improvement and patient safety programs.	3. Resident participation in hospital-wide interprofessional RCAs with formal process for distribution of information to the residency program<br>4. Hospital-wide GME QIPS Committee involvement (resident run, specifically PS events that cross specialties discussed) & formal process for distributing the information to the residency program<br><br>**Outcomes Measurement**<br>1. % Attendance at PS Didactics<br>2. % Residents participating on PS committees<br>3. % Residents involved in hospital RCAs
PS Pathway 3: Culture of safety<br>A culture of safety requires a preoccupation with identification of vulnerabilities and a willingness to transparently deal with them. To this end, the safety system is perceived as fair and effective in bringing about needed improvements. The organization has formal mechanisms to assess attitudes toward safety and improvement in order to identify areas requiring intervention.<br><br>**Properties include:**<br>● Residents/fellows and faculty members perceive that the clinical site provides a supportive culture for reporting patient safety	**ABP Content Specifications**<br>**Key principles of patient safety (cont…)**<br>3. Understand the importance of leadership in creating a culture of safety in the health-care system	**Educational Modality**<br>1. QI Project Simulation Workshop: Increasing Resident Reporting of PS events (Identify barriers, Educate on Importance, Educate on what/how to report, Develop feedback mechanism to those reporting, Review support for those involved in PS events)<br><br>**Outcomes Measurement**<br>1. Clinical Site Culture of Safety Survey

events. <u>*The focus will be on the extent to which individuals perceive a culture that is supportive of reporting.*</u><br>● The clinical site has mechanisms to provide emotional support to residents/fellows involved in patient safety events. <u>*The focus will be on the availability of support, and the proportion of residents/fellows who use (or perceive they could use) the mechanisms to access support.*</u><br>● The clinical site conducts culture of safety surveys with residents/fellows, and faculty and staff members. <u>*The focus will be on the progression from initial conduct of surveys through the analysis of results and implementation of actions to improve the culture.*</u>		Results
PS Pathway 4: Resident/fellow experience in patient safety investigations and follow-up<br>Feedback and experiential learning are essential to developing true competence in the ability to identify causes and institute sustainable systems-based changes to ameliorate patient safety vulnerabilities.<br><br>**Properties include:**<br>● Residents/fellows participate as team members in real or simulated interprofessional clinical site-sponsored patient safety investigations (such as root cause analyses or other activities that include analysis, as well as formulation and implementation of actions). <u>*The focus will be on the proportion and degree of resident/fellow involvement in site-sponsored investigations.*</u><br>● Residents/fellows can describe the disposition and actions resulting from the reporting of an event at the clinical site. <u>*The focus will be on identification of processes for providing residents/fellows with feedback on safety reports, and the proportion of individuals who are able to describe the outcomes resulting from reporting an event.*</u><br>● The clinical site provides feedback to residents/fellows on safety event reports and investigations. <u>*The focus will be on dissemination of lessons learned within programs and across the clinical site.*</u>	<u>**ABP Content Specifications**</u><br>**Methods to reduce medical adverse events (cont…)**<br>9. Understand and apply root cause analysis to determine the factors contributing to an error<br>10. Anticipate system vulnerabilities by applying failure mode effects analysis<br>11. Understand and apply evidence-based interventions to reduce medical adverse events<br>12. Use best-practice guidelines to reduce medical adverse events<br>13. Use effective methods of communication to reduce errors in the health-care setting<br>14. Understand and apply methodologies to prevent medication errors<br>**Key principles of patient safety (cont…)**<br>1. Apply knowledge of human factors in the design of systems and processes promoting patient safety<br>2. Promote effective team functioning in the prevention of medical error	<u>**Educational Modality**</u><br>1. Resident participation in hospital-wide interprofessional RCAs with formal process for distribution of information to the residency program<br>2. QI Project Simulation Workshop: Increasing Resident Reporting of PS events (Identify barriers, Educate on Importance, Educate on what/how to report, Develop feedback mechanism to those reporting, Review support for those involved in PS events)<br><br><u>**Outcomes Measurement**</u><br>1. % Residents Involved in Hospital RCAs<br>2. % Residents Completing Resident Peer Review of PS event<br>3. % Residents that obtained feedback on reported PS event
PS Pathway 5: Clinical site monitoring of resident/fellow engagement in patient safety<br>Residents/fellows are a vital component to the continual improvement of clinical care to patients; their participation in patient safety activities is essential.<br><br>**Properties include:**<br>● The clinical site monitors resident/fellow reporting of safety		<u>**Educational Modality**</u><br>1. Resident participation in hospital-wide interprofessional RCAs with formal process for distribution of information to the residency program<br>2. QI Project Simulation Workshop: Increasing Resident Reporting of PS

events. *<u>The focus will be on the progression from basic tracking of resident/fellow reporting to keeping the clinical site's governing body apprised of resident/fellow involvement in patient safety events, investigations, and resulting outcomes.</u>*<br>● Data from the monitoring process are used to develop and implement actions that improve patient care. *<u>The focus will be on the clinical site's usage of resident/fellow safety reports in developing and implementing improvements in patient safety.</u>*<br><br>***PS Pathway 6: Clinical site monitoring of faculty member engagement in patient safety***<br>Faculty members are a vital component to the continual improvement of clinical care to patients; their participation in patient safety activities is essential.<br><br>**Properties include:**<br>● The clinical site monitors faculty member reporting of safety events. *<u>The focus will be on the progression from basic tracking of faculty member reporting to keeping the clinical site's governing body and GMEC apprised of faculty member involvement in patient safety events, investigations, and resulting outcomes.</u>*<br>● Data from the monitoring process are used to develop and implement actions that improve patient care. *<u>The focus will be on the clinical site's usage of faculty safety reports in developing and implementing improvements in patient safety.</u>*		events (Identify barriers, Educate on Importance, Educate on what/how to report, Develop feedback mechanism to those reporting, Review support for those involved in PS events)<br>3. Hospital-wide GME PSQI Committee involvement (resident run, specifically PS events that cross specialties discussed) & formal process for distributing the information to the residency program<br>6. Resident Peer Review on Safety Events (each event is classified- ie preventable adverse event; abbreviated RCAs completed; recommendations made)<br><br>**<u>Outcomes Measurement</u>**<br>[illegible]. % Residents/faculty that know how to report PS events<br>[illegible]. % Residents/faculty who know their roles & responsibilities in reporting<br>[illegible]. % Residents/faculty that have reported safety events
PS Pathway 7: Resident/fellow education and experience in disclosure of events<br>Patient-centered care requires patients to be apprised of clinical situations which affect them. This is an important skill for physicians in residency/fellowship to develop and apply<br><br>**Properties include:**<br>● Residents/fellows receive hands-on training on how patient safety events are disclosed to patients and families at the clinical site. *<u>The focus will be on the proportion of residents/fellows receiving disclosure training, including participation in simulation activities, and whether the clinical site shares examples of best practices throughout the organization.</u>*<br>● Residents/fellows are involved in disclosure of patient safety events to patients and families at the clinical site. *<u>The focus will be on the proportion of residents/fellows involved in disclosure of patient safety events.</u>*	**<u>ABP Content Specifications</u>**<br>**Disclosure of medical errors**<br>1. Use appropriate means to disclose medical errors to patients<br>2. Apply appropriate methods of support for patients and their families after an error producing medical harm occur<br>3. Use appropriate methods of support for physicians and other health-care providers after an error producing medical harm occurs	**<u>Educational Modality</u>**<br>1. Disclosure training workshop<br><br>**<u>Outcomes Measurement</u>**<br>1. % Residents involved in disclosure of PS event to patient/ family

HQ Pathway 1: Education on quality improvement	**ABP Content Specifications**	**Educational Modality**
Formal educational activities that create a shared mental model with regard to health care quality-related goals, tools, and techniques are necessary in order for health care professionals to consistently work in a well-coordinated manner to achieve health care quality improvement goals.<br><br>**Properties include:**<br>● Residents/fellows receive progressive education and training on quality improvement that involves experiential learning. *The focus will be on the extent to which residents/fellows receive experiential training in quality improvement that includes consideration of underuse, overuse, and misuse in diagnosis or treatment of patients.*<br>● Residents/fellows and faculty members are engaged in quality improvement educational activities where the clinical site's systems-based challenges are presented, and techniques for designing and implementing systems changes are discussed. *The focus will be on the proportion of individuals who are engaged in quality improvement educational activities that include the above elements in their content.*<br>● Residents/fellows and faculty members are familiar with the clinical site's priorities for quality improvement. *The focus will be on the proportion of individuals familiar with the site's priorities, and the proportion of individuals aware of the site's progress and outcomes.*<br>● The clinical site's quality improvement education program is developed collaboratively by quality officers, residents/fellows, faculty members, nurses, and other staff members to reflect the clinical site's quality program's experience and goals. *The focus will be on the inclusion of GME leadership, residents/fellows, and faculty members in the process of developing the clinical site's quality education program and its dissemination throughout the organization, including to residents/fellows and faculty members.*<br>● Faculty members report that they are proficient in clinical quality improvement. *The focus will be on the proportion of faculty members that report proficiency in clinical quality improvement.*<br>● Residents/fellows are engaged in periodic quality improvement educational activities in which systems-based challenges are highlighted and approaches to designing and implementing system changes are discussed. *The focus will be on the proportion of residents/fellows engaged in quality improvement*	**Core principles of quality improvement**<br>a. Understand what a system is (eg, people, procedures, equipment) and how each component of that system affects outcome<br>b. Recognize that analysis of variation in data is critical in quality improvement to understand whether the variation is actually improvement<br>c. Understand that quality improvement is based on applying a scientific method to improving human systems<br>d. Apply the psychology of change (eg, motivating people to improve) to improve health-care systems<br>e. Recognize that quality improvement requires looking at data or processes (ie, trends) over time<br>f. Identify the components of the Langley Model for Improvement	1. IHI QI Modules<br>2. Workshops on applying QI methodology (process mapping, fishbone diagram, RCA, key driver diagram, The Model for Improvement)<br>3. Personal Improvement Projects used to learn and practice methodology<br>4. Participate in and lead group QI projects in the healthcare setting<br>5. QI Overview didactic lecture that includes hospital's specific QI priorities<br><br>**Outcomes Measurement**<br>1. % Residents familiar with Hospital's QI priorities<br>2. Faculty needs assessment, % proficient in clinical QI<br>3. % Residents completing QI Project through at least 2 PDSA cycles<br>4. % Resident QI projects presented at medical conferences

<u>educational activities around systems-based improvements.</u>		
HQ Pathway 2: Resident/fellow engagement in quality improvement activities<br>Experiential learning is essential to developing the ability to identify and institute sustainable systems-based changes to improve patient care.<br><br>**Properties include:**<br>● Residents/fellows are actively involved in the quality improvement activities at the clinical site. *<u>The focus will be on the proportion of residents/fellows that are: actively involved in a quality improvement project at the site; involved in interprofessional teams, focused on measures of resource use, aligned and integrated with the clinical site's priorities; and involved in site-wide initiatives with active oversight by the clinical site's quality improvement leadership.</u>*		**<u>Educational Modality</u>**<br>1. Participate in and lead group QI projects in the healthcare setting<br>2. QI Overview didactic lecture that includes hospital's specific QI priorities<br><br>**<u>Outcomes Measurement</u>**<br>1. % Residents completing QI Project through at least 2 PDSA cycles<br>2. % Residents participating on PS/QI committees
HQ Pathway 3: Residents/fellows receive data on quality metrics<br>Access to data is essential to prioritizing activities for care improvement and evaluating success of improvement efforts.<br><br>**Properties include:**<br>Residents/fellows receive, from the clinical site, specialty-specific data on quality metrics and benchmarks related to their patient populations. *<u>The focus will be on the proportion of residents/fellows receiving patient data, and on the level of data specificity (e.g., aggregated clinical site data versus data specific to a resident's/fellow's patient population).</u>*	**<u>RRC PS/QI Guidelines</u>**<br>**Practice Based Learning and Improvement**<br>IV.A.5.c) Residents must demonstrate the ability to investigate and evaluate their care of patients, to appraise and assimilate scientific evidence, and to continuously improve patient care based on constant self-evaluation and life-long learning. Residents are expected to develop skills and habits to be able to meet the following goals:<br>● identify strengths, deficiencies, and limits in one's knowledge and expertise;<br>● set learning and improvement goals;<br>● identify and perform appropriate learning activities;<br>● systematically analyze practice using quality improvement methods, and implement changes with the goal of practice improvement;<br>● incorporate formative evaluation feedback into daily practice;<br>● locate, appraise, and assimilate evidence from scientific studies related to their patients' health problems;<br>● take primary responsibility for lifelong learning to improve knowledge, skills, and practice performance through familiarity with general and experience-specific goals and objectives and attendance at conferences.<br>VI.A.6. Residents and faculty members must demonstrate an	**<u>Educational Modality</u>**<br>1. Resident lecture on their patient panels from which reports can be run<br><br>**<u>Outcomes Measurement</u>**<br>1. % Residents creating a report on clinical outcomes of their patient panel

	understanding and acceptance of their personal role in the following:<br>attention to lifelong learning; and the monitoring of their patient care performance improvement indicators.	
HQ Pathway 4: Resident/fellow engagement in planning for quality improvement<br>In order to understand quality from a systems-based perspective, it is necessary to be familiar with the entire cycle of quality improvement (QI) from planning through execution and reassessment.<br><br>**Properties include:**<br>● Residents/fellows participate in departmental and clinical site-wide QI committees. *The focus will be on resident/fellow participation on the clinical site's QI committees, from department-level committees to committees of the governing body.*<br>● The clinical site monitors resident/fellow efforts in QI. *The focus will be on basic tracking of resident/fellow involvement in QI, keeping the clinical site's governing body and GMEC apprised of resident/fellow involvement, and developing site-specific strategies to maximize resident participation.*	**MOC Part IV Specs**<br>● Clearly defined problem statement that links to 1 or more of the Institute of Medicine dimensions of quality care<br>● Specific aim statement (measurable, time bound)<br>● Balanced set of clearly defined measures (process, outcome, and balancing)<br>● A data collection plan that accounts for data integrity and includes a sound sampling strategy<br>● Project interventions that have been thoughtfully chosen on the basis of an in-depth understanding of the problem and the potential barriers to change<br>● Data for each measure displayed in run or statistical process control charts and a plan for how the data will be used to drive improvement<br>● A plan for sharing the results with both the clinicians engaged in the project and the key stakeholders in the institution	**Educational Modality**<br>1. IHI Modules<br>2. Workshops on QI Methodology<br>3. Group QI projects<br>4. Resident participation on hospital-wide interprofessional PS/QI committees with formal process for distribution of information to the residency program<br><br>**Outcomes Measurement**<br>1. % Residents completing QI Project through at least 2 PDSA cycles<br>2. % Residents participating on PS/QI committees
HQ Pathway 5: Resident/fellow and faculty member education on reducing health care disparities<br>Formal educational activities that create a shared mental model with regard to health care quality-related goals, tools, and techniques are necessary for health care professionals to consistently work in a well-coordinated manner to achieve a true patient-centered approach that considers the variety of circumstances and needs of individual patients<br><br>**Properties include:**<br>● Residents/fellows and faculty members receive education on identifying and reducing health care disparities relevant to the patient population served by the clinical site. *The focus will be on the extent to which individuals receive education on the clinical site's priorities and goals for addressing health care disparities in its patient population.*<br>● Residents/fellows and faculty members receive training in cultural competency relevant to the patient population served by the clinical site. *The focus will be on the extent to which individuals receive training in cultural competency relevant to the patient population served by the clinical site.*<br>● Residents/fellows and faculty members know the clinical site's		**Educational Modality**<br>1. QI Overview didactic lecture includes hospital's specific priorities for addressing health care disparities<br>2. Resident lecture on their patient panels from which reports can be run and stratified by social determinants of health<br><br>**Outcomes Measurement**<br>1. % Residents familiar with Hospital's priorities for addressing health care disparities<br>2. % Residents creating a report on clinical outcomes of their patient panel stratified by social determinants of health

priorities for addressing health care disparities. <u>*The focus will be on the proportion of individuals able to describe the site-specific priorities for addressing health care disparities, and the proportion that are aware of the clinical site's progress in meeting its goals to address the priorities.*</u>		
HQ Pathway 6: Resident/fellow engagement in clinical site initiatives to address health care disparities<br>Experiential learning is essential to developing the ability to identify and institute sustainable systems-based changes to address health care disparities.<br><br>**Properties include:**<br>● Residents/fellows are engaged in QI activities addressing health care disparities for the vulnerable populations served by the clinical site. <u>*The focus will be on the proportion of programs that involve residents/fellows in QI projects to reduce health care disparities, as well as on assessing whether there is some resident/fellow engagement in clinical site initiatives to address health care disparities, and resident/fellow engagement with the clinical site in defining priorities and strategies to address health care disparities specific to the site's patient population.*</u>		<u>**Educational Modality**</u><br>1. QI Overview didactic lecture includes hospital's specific priorities for addressing health care disparities<br>2. Resident lecture on their patient panels from which reports can be run and stratified by social determinants of health<br><br><u>**Outcomes Measurement**</u><br>. % Residents familiar with Hospital's priorities for addressing health care disparities<br>. % Resident QI projects in which results are stratified by social determinants of health
	REFERENCES	
Accreditation Council for Graduate Medical Education, CLER PATHWAYS TO EXCELLENCE – PATIENT SAFETY AND QUALITY, 2014. https://www.acgme.org/acgmeweb/Portals/0/PDFs/CLER/CLER_Brochure.pdf	*The American Board of Pediatrics, Content Outline General Pediatrics https://www.abp.org/content/general-pediatrics-content-outline*<br><br>*Residency Review Committee, Common Program Requirements (Residency), 2013.*<br><br>*Table 5: Craig, M, et.al, Pediatric Resident Education in QI: A National Survey. Academic Peds 2014; 14:54-61*	

Requirements, recommendations, and guidelines were gathered and cross tabulated. When content fit multiple themes, the best fit was chosen.

Supplemental Table 2: Patient and Process Related Outcomes as a Result of Resident QI Projects

Improved Patient-Centered Outcomes
• HPV vaccination rates among adolescents<br>• Caregiver understanding of their child's eczema diagnosis and treatment plan<br>• Reported overall treatment effectiveness in children with eczema<br>• Patients with obesity who maintained or decreased their BMI percentiles<br>• Families with food insecurity who received food
Improved clinical healthcare processes
• Caregiver reminders for overdue vaccines via RN calls<br>• Completion of medication histories within two hours of admission<br>• Completion of discharge instructions for transitions from hospital to home<br>• Standardization of a stepwise eczema treatment plan based on severity via a pictorial eczema action plan<br>• Communication between residents and nurses via a standard text paging system utilizing a priority scale<br>• Completion of asthma action plans including demonstration of the correct technique for using medications<br>• Developing a safe sleep bundle for hospitalized infants<br>• Screening for adverse childhood events<br>• Accurate patient status assignments on admission (inpatient versus observation)<br>• Standardized obesity follow up visits<br>• Shift handoffs using the IPASS format and minimizing distractions and interruptions<br>• Hospitalized infants following the American Academy of Pediatrics safe sleep guidelines<br>• Completion of family centered rounds<br>• Screening for food insecurity and providing resources<br>• Geographic-based teams for enhanced care team communication
